# Recognition of a core fragment of *Beauveria bassiana* hydrophobin gene promoter (P *hyd1*) and its special use in improving fungal biocontrol potential

**DOI:** 10.1111/j.1751-7915.2012.00351.x

**Published:** 2012-05-28

**Authors:** Zheng-Liang Wang, Sheng-Hua Ying, Ming-Guang Feng

**Affiliations:** Institute of Microbiology, College of Life Sciences, Zhejiang UniversityHangzhou, Zhejiang, 310058, China

## Abstract

To identify a suitable promoter for use in engineering fungal entomopathogens to improve heterologous gene expression and fungal biocontrol potential, a 1798 bp promoter (P*hyd1*) upstream of *Beauveria bassiana* class I hydrophobin gene (*hyd1*) was optimized by upstream truncation and site-directed mutation. A truncated 1290 bp fragment (P*hyd1-t1*) drove *eGFP* expression in *B. bassiana* much more efficiently than full-length P*hyd1*. Further truncating P*hyd1-t1* to 1179, 991 and 791 bp or mutating one of the binding domains of three transcription factors in P*hyd1-t1* reduced significantly the expression of eGFP (enhanced green fluorescence protein). Under P*hyd1-t1* control, eGFP was expressed more abundantly in conidiogenic cells and conidia than in mycelia. Therefore, P*hyd1-t1* was used to integrate a bacterium-derived, insect midgut-specific toxin (vip3Aa1) gene into *B. bassiana*, yielding a transgenic strain (BbHV8) expressing 9.8-fold more toxin molecules in conidia than a counterpart strain (BbV28) expressing the toxin under the control of P*gpdA*, a promoter widely used for gene expression in fungi. Consequently, BbHV8 showed much higher *per os* virulence to *Spodoptera litura* larvae than BbV28 in standardized bioassays with normal conidia for both cuticle penetration and ingestion or heat-killed conidia for ingestion only. Conclusively, P*hyd1-t1* is a useful tool for enhancing beneficial protein expression, such as vip3Aa1, in fungal conidia, which are the active ingredients of mycoinsecticides.

## Introduction

Entomopathogenic fungi, such as *Beauveria bassiana* and *Metarhizium anisopliae*, are fungal biocontrol agents widely applied to arthropod pest control (Feng *et al*., 1994; Roberts and St Leger, 2004). Candidate strains for commercial development are usually selected for their high virulence to target pests and tolerance to high temperature (Rangel *et al*., 2005; Li and Feng, 2009), solar UV irradiation (Braga *et al*., 2001; Huang and Feng, 2009) and fungicide sprays often encountered for plant disease control in the field (Zou *et al*., 2006; Song *et al*., 2012). However, not all desired traits exist in a candidate strain. This makes it necessary to engineer genetically the fungal candidate for more improved traits.

Fungal genetic improvement requires a powerful promoter to drive steadily replicable expression of target genes in a host strain (Ruiz-Diez, 2002). A widely applied fungal promoter is P*gpdA*, i.e. the promoter of *gpdA* gene encoding *Aspergillus nidulans* glyceraldehyde-3-phosphate dehydrogenase. This promoter enables to drive the expression of various heterologous genes in filamentous fungi (Brakhage *et al*., 1999). Other fungal promoters identified include those that activate the genes of *Aspergillus* alcR and glaA (Punt *et al*., 1995; Santerre-Henriksen *et al*., 1999; Mathieu *et al*., 2000), *Pseudozyma flocculasa* actin (Neveu *et al*., 2007) and *Penicillium funiculosum* histone H4 (Belshaw *et al*., 2002). Two promoters that regulate the transcriptional expressions of *B. bassiana gpd* (Liao *et al*., 2008) and *M. anisopliae* Tef-1α gene (Nakazato *et al*., 2006) have also been reported. However, none of the fungal promoters is known to drive the expression of beneficial genes specifically in fungal conidia, which are usually the active ingredients of mycoinsecticides (de Faria and Wraight, 2007).

Hydrophobins are small amphipathic proteins ubiquitously present in filamentous fungi and are involved in a variety of biological functions, such as host attachment, pathogenesis, fruit body formation and sporulation (Wösten, 2001). A 188 bp core fragment of cryparin gene promoter of *Cryphonectria parasitica* (Kim *et al*., 2008) has been found to drive high expression of heterologous proteins in the same fungus (Kwon *et al*., 2009). A gene encoding *B. bassiana* class I hydrophobin (*hyd1*) was expressed well in almost all developmental stages of the entomopathogenic fungus (Cho *et al*., 2007). The previous studies hint that the *hyd1* promoter region might harbour a core fragment to regulate heterologous protein expression specifically during conidiophore development and conidiation by fungal engineering.

This study sought to identify the core fragment by analysing the full-length 1798 bp promoter (P*hyd1*) and the binding domains of its transcription factors (TFs) through upstream truncation and site mutation. The site-mutated binding domains included the common DNA-binding motif of the basic helix–loop–helix regulator StuA (WCGCGWNM), the conserved domain of the high-mobility-group box factor Mat-Mc (YCNATTGTYW) and the single zinc finger DNA-binding motif (Cys-X2-Cys-X17-Cys-X2-Cys type) of the regulator NIT_2_ (TATCTM). These TFs are known to regulate gene expression in fungi through interaction with specific DNA-binding motifs in promoter regions (Dutton *et al*., 1997; Kjaerulff *et al*., 1997; Feng and Marzluf, 1998; Scherer *et al*., 2002). The full-length P*hyd1* and four truncated and three site-mutated fragments of it were compared with the well-known promoter P*gpdA* for their efficiencies in driving the expression of enhanced green fluorescence protein (eGFP) in *B. bassiana*. An optimized fragment was used to enhance the fungal biocontrol potential by engineering *B. bassiana* with a gene encoding the insecticidal protein vip3Aa1 (an insect midgut-acting toxin from *Bacillus thuringiensis*) for high expression in transgenic conidia.

## Results

### Features of the P*hyd1* promoter

A flanking sequence upstream of the initial codon (ATG) of the *hyd1* gene (Cho *et al*., 2007) was amplified as a 1798 bp fragment (GenBank ID: GU936631) from the genomic DNA of *B. bassiana* ARSEF 2860 (Bb2860 herein) using paired primers Phyd1-F/R (Table 1). Online sequence analysis indicated the locations of initial transcription site at −106 bp, typical TATA box (TATAAA) at −24 bp, CAAT box at −92 bp and a 15 bp C/T-rich region at −85 bp. The binding domains of the three TFs StuA (GCTCGCGAGC), NIT_2_ (TATCTA) and Mat-Mc (TCGATTGTCT) were located at −201, −626 and −1066 bp respectively.

**Table 1:** The primers designed for gene manipulation

Paired primers	Sequences (5′–3′)^a^	Purpose
Hyd1-F/R	ATGCGTTTCGCTCTTGCCATCAC/TTACTGGATAAGACTGCCAATGG	Cloning *hyd1*
Phyd1-F/R	GGAAGATCTAATTAGTCAGGCACCCTTGACGC/CATGCCATGGATGACGGTATTGTTTATTTGGTTG	Cloning P*hyd1*
Phyd1-t1F/R	GGAAGATCTCGTGCCAGTTTCTCCAAGCAACTAC/CATGCCATGGATGACGGTATTGTTTATTTGGTTG	Cloning P*hyd1-t1*
Phyd1-t2F/R	GGAAGATCTTGTCTCTCTTTTTTTATTGTATCT/CATGCCATGGATGACGGTATTGTTTATTTGGTTG	Cloning P*hyd1-t2*
Phyd1-t3F/R	GGAAGATCTCCTGAAAAGGTTTTATTGCGGC/CATGCCATGGATGACGGTATTGTTTATTTGGTTG	Cloning P*hyd1-t3*
Phyd1-t4F/R	GGAAGATCTCAACATCAGCAGTAGATGGGCGGCT/CATGCCATGGATGACGGTATTGTTTATTTGGTTG	Cloning P*hyd1-t3*
StuA-1/2	GCAAGATATGCATGACGTAGCATTAAGCTTGCAATGCGGAAAGAAATGCACAAGTC/GACTTGTGCATTTCTTTCCGCATTGCAAGCTTAATGCTACGTCATGCATATCTTGC	Mutating StuA domain
Mat-1/2	CGACAATGCGTCTGGCTCATACCAAGCTTGCGTGCTGGATTGCATCAGATG/TCATCTGATGCAATCCAGCACGCAAGCTTGGTATGAGCCAGACGCATTGTC	Mutating Mat-Mc domain
NIH-1/2	CTCGCTACACATCGGTCGCCTGCAAGCTTAATTATGTTATCTACCATCCGAAT/CGCGAGCAATGCTACGTCATGCAAAGCTTGCTCCTTTGGGCGATGGTGGTGT	Mutating NIT_2_ domain
Bar-F/R	AGAACGACGCCCGGCCGACAT/CTGCCAGAAACCCACGTCATGC	Identifying transformants
eGFP-F/R	ATGGTGAGCAAGGGCGAGGAGCTG/GGACTTGTACAGCTCGTCCATGCC	Identifying transformants
Vip-F/R	CCTTCAGCAACCCGAACTACGC/GCTCGCGCAGGTAGCTCTTACAG	Identifying transformants
qVip-F/R	GCATCAAGTACGTGAACGAGAAG/GTCGTGGAAGGTGTTCAGGTAG	qRT-PCR for transcripts
q18S-F/R	TGGTTTCTAGGACCGCCGTAA/CCTTGGCAAATGCTTTCGC	qRT-PCR for transcripts

a The underlined regions denote the introduced cleavage sites of *Bgl*II and *Nco*I enzymes for a P*hyd1* truncation or the substitute of *Hin*dIII restriction site for the site mutation of a TF-binding domain.

### Core fragment of P*hyd1*

**Fig. 1:**
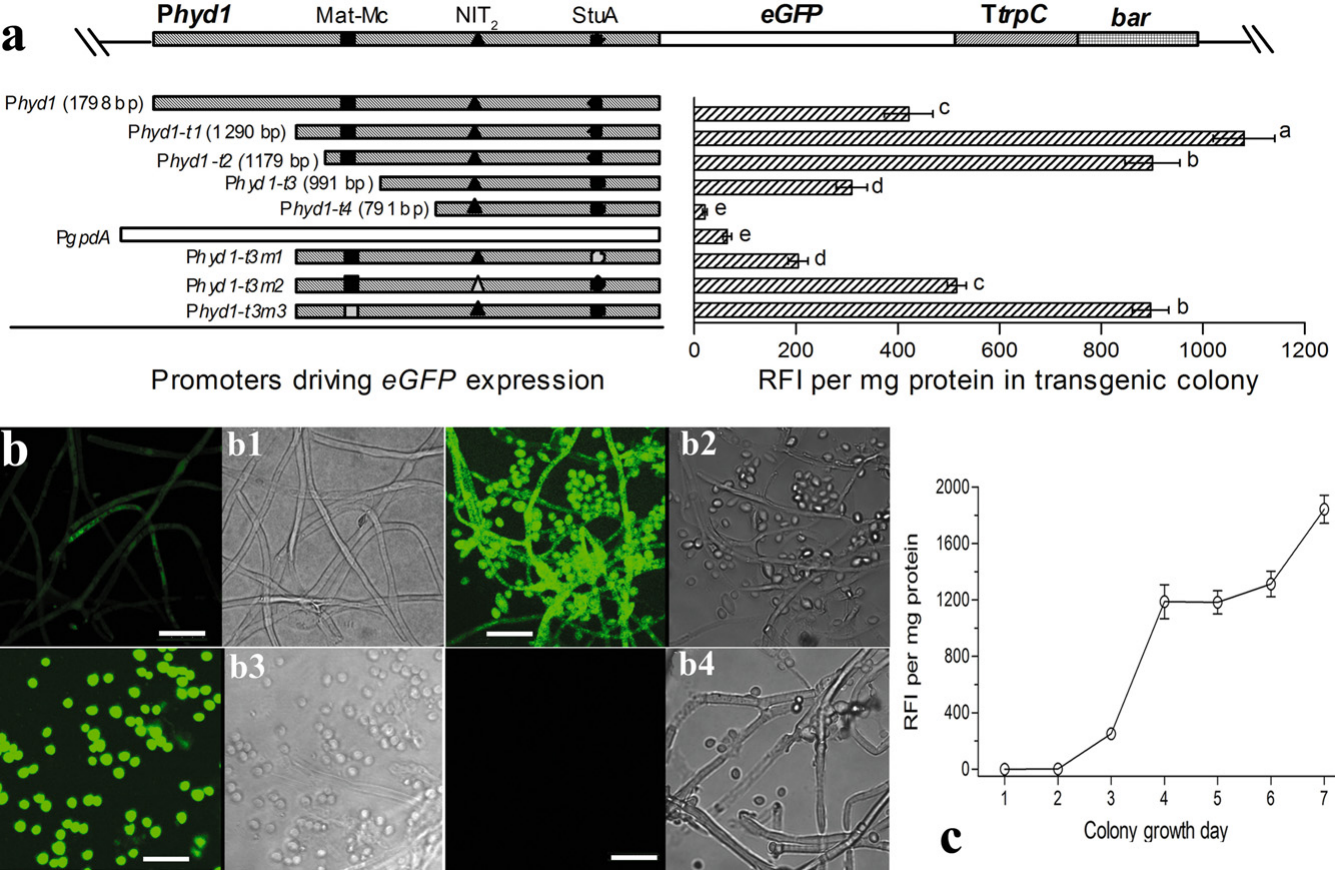
Recognition of a core fragment in the *hyd1* promoter region (P*hyd1*) of *B. bassiana*. a. Concatenation of expression elements in binary plasmids for insertion into wild-type strain (Bb2860) and comparison of eGFP expression levels (RFI) in the 4 day colonies of fungal transformants controlled by full-length, four truncated and three site-mutated fragments of P*hyd1* and the widely used P*gpdA* respectively. Close and open symbols denote the normal and mutated binding domains of StuA (square), Mat-Mc (triangle) and NIT_2_ (circle) respectively. Error bars: SD of the mean of three transformants. b. Laser confocal fluorescence (*left*) and bright (*right*) images of samples of an eGFP-expressing colony (b1−b3: grown at 25°C for 2, 4 and 7 days respectively under P*hyd1-t1* control; b4: the same images of a fungal mass from 4 day wild-type colony). Scale bars: 10 μm. c. RFI trend during the 7 day growth of three P*hyd1-t1* controlled transformants on SDAY plates at 25°C.

The core fragment of P*hyd1* was located using four truncated and three site-mutated P*hyd1* fragments to drive *eGFP* expression in transgenic Bb2860. Relative fluorescence intensities (RFI) under their controls (Fig. 1a) differed significantly (*F*_8,18_ = 114, *P* < 0.0001 in one-way analysis of variance). The full-length P*hyd1* was capable of driving *eGFP* expression but its efficiency was 1.6-fold lower than the P*hyd1-t1* counterpart. This truncated fragment resulted in maximal RFI (1081 ± 104) in transgenic colonies. This RFI was 15.6-fold higher than that from the P*gpdA*-controlled transformants. However, further truncating P*hyd1-t1* (1290 bp) to P*hyd1-t2* (1179 bp), P*hyd1-t3* (991 bp) and P*hyd1-t4* (791 bp) reduced the *eGFP* expression by 16.7%, 71.3% and 98% respectively. All site mutations in P*hyd1-t1* also led to significant RFI reductions in the transgenic colonies (Fisher's least significant difference, *P* < 0.05). The net RFI reductions were 17%, 52% and 81% for the mutated binding domains of Mat-Mc, NIT_2_ and StuA respectively. Apparently, P*hyd1-t1* vectoring the essential binding domains of the three TFs was the core promoter fragment to maximize heterologous gene expression in *B. bassiana*.

**Fig. 2:**
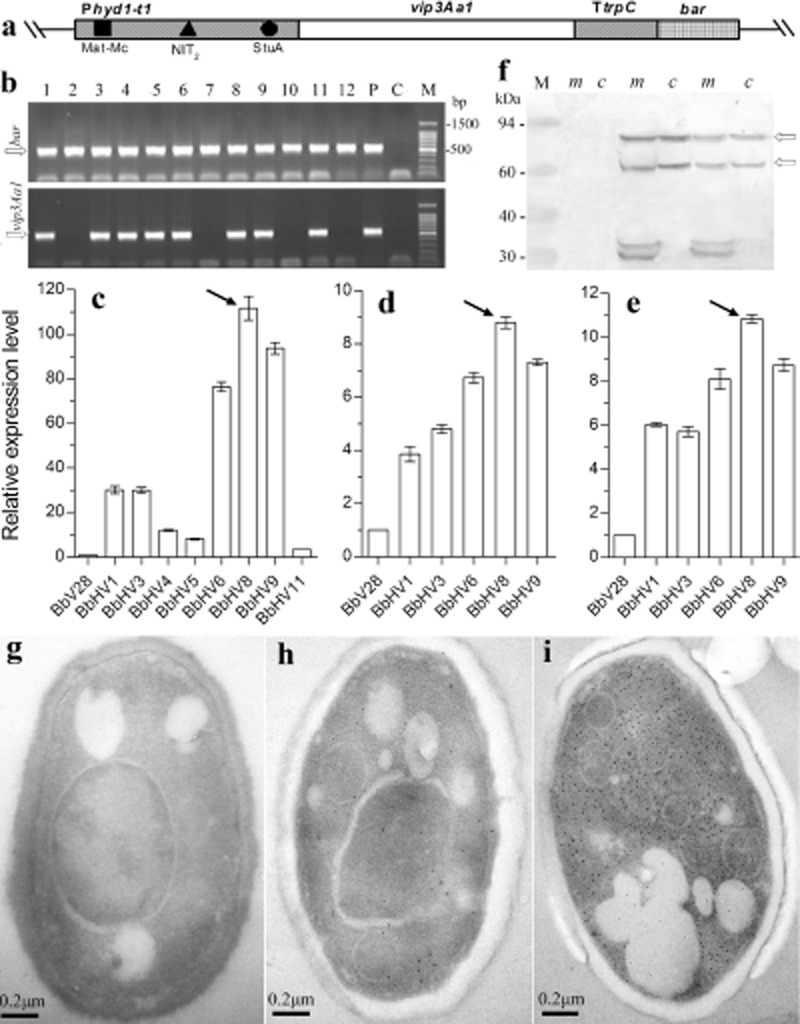
Overexpression of vip3Aa1 in *B. bassiana* strains engineered under P*hyd1-t1* control. a. Concatenation of expression elements in binary plasmid constructed for transformation.b. PCR detection for the presence of *vip3Aa1* in 12 transformants (lanes 1−12). P, BbV28; C, wild-type (Bb2860).c. Relative transcript levels of *vip3Aa1* in 4 day colonies of eight positive transformants (under P*hyd1-t1* control) versus P*gpdA*-controlled BbV28 in qRT-PCR. d and e. Relative expression levels of vip3Aa1 in the mycelia and conidia of five selected transformants versus BbV28 in elisa respectively. f. Western blots of mycelial (*m*) and conidial (*c*) extracts of Bb2860 (left), BbHV8 (middle) and BbV28 (right) probed by the polyclonal antibody of vip3Aa1. g–i. Immunogold localization of vip3Aa1 molecules expressed in the conidia of Bb2860, BbV28 and BbHV8 respectively. Note that 10 nm colloidal gold particles labelled with the polyclonal antibody and goat anti-rabbit IgG antibody are much denser in BbHV8 (i) than in BbV28 (h) but absent in Bb2860 (g).

Interestingly, the *eGFP* expression under P*hyd1-t1* control was barely detected during the first 48 h of incubation of selected transformants on Sabouraud dextrose agar plus yeast extract (SDAY) at 25°C but rapidly increased following this time period. The eGFP-emitted fluorescence under laser confocal microscope was much more intense in conidiogenic cells and conidia than in younger mycelia (Fig. 2b). Mean RFI in the colonies increased from 2.4 on day 2 to 1187 on day 4 and reached a peak of 1842 on day 7 (Fig. 2c), when conidiation was completed. These data indicated that P*hyd1-t1* enabled the activation of gene expression specifically during the conidiophore development and conidiation of *B. bassiana*.

### Overexpression of vip3Aa1 under P*hyd1-t1* control

The binary plasmid carrying the *vip3Aa1* and P*hyd1-t1* elements (Fig. 2a) was integrated into the competent blastospores of Bb2860. Eight positive transformants were identified from 12 colonies grown on the selective plates through PCR analysis (Fig. 2b). Quantitative real-time PCR (qRT-PCR) assays (Fig. 2c) indicated that the transcript levels of the toxin gene in their colonies grown for 4 days on SDAY plates at 25°C were enhanced by 3.4- to 112-fold compared with the transcript of the same gene in BbV28 under P*gpdA* control. Moreover, enzyme-linked immunosorbent assays (elisa) indicated that the amounts of target toxin produced in the mycelia (Fig. 2d) and conidia (Fig. 2e) of five transformants selected in qRT-PCR was enhanced by 2.9- to 7.8-fold and 4.7- to 9.8-fold respectively. Of those, BbHV8 was selected as the best transformant due to its maximal levels in both gene transcription and protein expression.

Mature and active vip3Aa1 forms (∼ 88 and ∼ 62 kDa respectively) were detected in the mycelial and conidial extracts of BbHV8 and BbV28 by Western blotting with the vip3Aa1 antibody (Fig. 2f). In immunogold localization, the mean (± SD) densities of 10 nm colloidal gold particles on the five ultrathin sections of Bb2860, BbV28 and BbHV8 conidia (Fig. 2g−i) labelled by the rabbit antibody and goat anti-rabbit IgG antibody were estimated as 0, 21 (± 4) and 168 (± 17) particles per square micrometre respectively. The labelled vip3Aa1 density in BbHV8 conidia was eightfold of that in BbV28, well in agreement with the elisa estimates.

### Virulence of strains to *Spodoptera litura* larvae

**Fig. 3:**
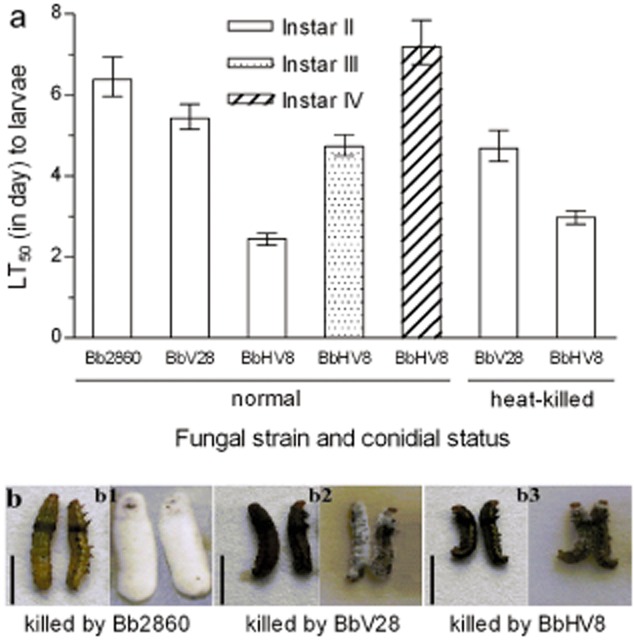
a. LT_50_s of Bb2860 (wild-type), BbV28 (under P*gpdA* control) and BbHV8 (under P*hyd1-t1* control) against *S. litura* larvae (error bars: 95% CIs). b. Symptoms of cadavers died of Bb2860 (b1), BbV28 (b2) and BbHV8 (b3). Left: fresh cadavers. Right: fungal outgrowths at saturated humidity. Scale bars: 5 mm.

In bioassays with normal conidial suspensions of BbHV8, BbV28 and Bb2860, standardized fungal sprays (2 × 10^7^ conidia per spray) resulted in the mean (± SD) deposit of 391 (± 20) conidia per square millimetre onto both *S. litura* larvae (for cuticle infection) and lotus leaf discs (for their ingestion). In the probit analyses of time-mortality trends, the median lethal time (LT_50_) and associated 95% confidence interval (CI) against the second-instar larvae were estimated as 6.4 (6.0−6.9) days for Bb2860 (Pearson's *χ*^2^ = 6.02, d.f. = 5, *P* = 0.30), 5.4 (5.1−5.8) days for BbV28 (*χ*^2^ = 0.60, d.f. = 5, *P* = 0.99) and 2.5 (2.3−2.6) days for BbHV8 (*χ*^2^ = 0.40, d.f. = 3, *P* = 0.94), as shown in Fig. 3a. The same estimates of BbHV8 against instars III and IV were 4.7 (4.5−5.0) days (*χ*^2^ = 10.12, d.f. = 6, *P* = 0.12) and 7.2 (6.8−7.8) days (*χ*^2^ = 2.21, d.f. = 4, *P* = 0.70) respectively. In contrast, neither Bb2860 nor BbV28 had computable LT_50_ against later stage larvae because the two strains killed only 22% and 37% of third-instar larvae on day 8 and even fewer of fourth-instar larvae. Moreover, fungal outgrowths as a typical symptom of mycosis occurred heavily on all larvae that died of Bb2860 and sparsely on those that died of BbV28, but were absent on those killed by BbHV8 after 3−5 day incubation at saturated humidity (Fig. 3b). These data indicate that BbHV8 not only killed the younger larvae more rapidly than BbV28 and Bb2860 but also showed high killing activity on later stage larvae. The enhanced insecticidal activity of BbHV8 to *S. litura* larvae was likely attributable to the ingestion of more toxin molecules expressed in conidia.

Interestingly, this assumption was confirmed in the bioassays with the heat-treated conidia of the three strains. After exposure to 42°C for 3 h, all suspension samples contained no viable conidia after 24 h incubation on SDAY plates at 25°C. Fed with leaf discs harbouring 1394 (± 28) heat-killed conidia per square millimetre, the second-instar larvae of *S. litura* suffered from 100% and 66.7% moralities caused by BbHV8 and BbV28 on day 6 respectively, whereas no substantial mortality was attributed to the wild-type strain. No fungal outgrowths were found on all cadavers. The LT_50_s and associated 95% CIs (Fig. 3a) were estimated as 3.0 (2.8−3.1) and 4.7 (4.4−5.1) days for BbHV8 (*χ*^2^ = 2.72, d.f. = 4, *P* = 0.61) and BbV28 (*χ*^2^ = 8.59, d.f. = 5, *P* = 0.13) respectively. These data indicated that the ingestion of non-viable conidia harbouring more vip3Aa1 toxin molecules resulted in faster and more frequent mortality of the larvae.

## Discussion

As presented above, P*hyd1-t1* has proved to be the core fragment of the *hyd1* gene promoter of *B. bassiana*. It harbours the essential binding domains of the TFs StuA, Mat-Mc and NIT_2_ as well as a C/T-rich region, which exist in other fungal promoters and are crucial to initiating gene transcription (Toda *et al*., 2001; Bertossa *et al*., 2004). The full-length P*hyd1* was much less efficient in driving *eGFP* expression than the truncated core fragment (Fig. 1a), suggesting possible existence of negative repressive element(s) in the 508 bp region upstream of P*hyd1-t1*. The fact that truncating P*hyd1-t2* to P*hyd1-t3* caused 66% RFI reduction in transgenic colonies may indicate that a positive regulatory element might exist in the 189 bp region upstream of P*hyd1-t3*. Mutating the StuA binding domain with a *Hin*dIII restriction site reduced drastically eGFP expression by 80%, an indication that this domain may be crucial to the core fragment. A 51% RFI decrease caused by the mutation of the NIT_2_ binding domain also consolidates its importance in regulating the gene expression.

Moreover, the temporal and spatial pattern of *eGFP* expression under P*hyd1-t1* control (Fig. 1b and c) has clarified the ability of this core promoter fragment to drive heterologous gene expression specifically during the conidiophore development and conidiation of *B. bassiana*. This supports a previous conclusion that hydrophobin-coding genes are usually transcribed at the stages of fruit body formation and sporulation (Wösten, 2001) and is also in agreement with the steady increase of *hyd1* expression in *B. bassiana* colonies during a growth period of 3−28 days at 26°C (Cho *et al*., 2007).

Like the eGFP expression pattern, the insect intestine toxin vip3Aa1 was expressed much more abundantly in the conidia than in the mycelia of BbHV8, which was engineered under P*hyd1-t1* control. Compared with BbV28 engineered with the same toxin gene under P*gpdA* control (Qin *et al*., 2010), BbHV8 produced nearly 10-fold more toxin molecules in conidia and thus killed *S. litura* larvae more rapidly and effectively irrespective of normal or heat-killed conidia ingested (Fig. 3a). Due to the alkaline midgut environment of *S. litura* (Skibbe *et al*., 1996), BbV28 conidia were shown to release the active form of the vip3Aa1 toxin into the midguts of *S. litura* larvae soon after ingestion (Qin *et al*., 2010). Theoretically, transgenic conidia harbouring more midgut-specific toxin molecules could release a greater quantity into larval midgut to achieve higher insecticidal activity after ingestion and appears to support the acceleration of BbHV8 killing action due to its higher titer of toxin.

Furthermore, P*hyd1-t1* is a small promoter (only 1290 bp) compared with the constitutive *gpdA* gene promoter (2200 bp) widely used in heterologous gene expression in fungi (Punt *et al*., 2002). Due to its capability of driving the expression of eGFP and vip3Aa1 genes much more efficiently during conidiation than P*gpdA*, P*hyd1-t1* is of great potential for use in engineering fungal biocontrol agents to improve their virulence to target pests and tolerance to environmental stresses, thereby facilitating the development of more efficacious and field-persistent mycoinsecticides.

Finally, although there is always a concern about environmental safety for the field application of genetically engineered fungi, we think that environmental risk rising from the use of transgenic *B. bassiana* strains expressing vip3A toxins under real conditions would be minimized for the following reasons. First, dozens of *B. bassiana* formulations have been registered for global pest control (de Faria and Wraight, 2007) without safety problems documented. Second, vip3A toxins act specifically on caterpillar midgut cells (Yu *et al*., 1997) and are safe to vertebrates (Brake *et al*., 2005; Peng *et al*., 2006). Transgenic crops expressing vip3A toxins have been permitted for field release (Llewellyn *et al*., 2007). Third and more interestingly, the new transgenic strain could not grow out of the caterpillars that rapidly died of conidial ingestion (Fig. 3b), suggesting a much less chance for its survival in the field and increasing its potential for commercial development. Nonetheless, it is essential to evaluate strictly its environmental safety before field release, warranting further study in the future.

## Experimental procedures

### Microbial strains and culture media

The wild-type strain *B. bassiana* ARSEF 2860 from the RW Holley Center for Agriculture and Health (Ithaca, NY, USA) was used as a recipient in gene transformation and maintained at 4°C on the slants of Sabouraud dextrose agar plus 1% yeast extract (SDAY). *Escherichia coli* DH5α and *E. coli* BL21(DE3) from Invitrogen (Shanghai, China) were cultured at 37°C in Luria–Bertani medium plus ampicillin (100 μg ml^−1^) or kanamycin (50 μg ml^−1^) depending on resistance type used for vector construction.

### Cloning and analysis of full-length P*hyd1* region

The *hyd1* gene was amplified from the extracted genomic DNA of Bb2860 in 50 μl reaction system via PCR (35 cycles of 30 s at 94°C, 30 s at 60°C and 30 s at 72°C) with paired primers Hyd1-F/R (Table 1), which were designed in terms of the *hyd1* open-reading frame (GenBank ID: EF452344). Its upstream region was obtained by two runs of DNA walking with SpeedUp™ Premix Kit II from Neuro-Hemin Biotec (Hangzhou, China). This putative promoter region was analysed online by Motif Search (http://motif.genome.jp) and tfsearch (http://www.Cbrc.jp/research/db/TFSEARCH.html) to locate binding domains of possible TFs as well as initial transcription site, TATA box and CAAT box. The full-length promoter sequence (P*hyd1*) was then amplified using the primers Phyd1-F/R (Table 1).

### Identification of a core fragment in P*hyd1*

Four truncated P*hyd1* fragments were amplified from the genomic DNA of Bb2860 using the common downstream primer Phyd1-R with *Nco*I site and the respective upstream primers Phyd1-t1F, Phyd1-t2F, Phyd1-t3F and Phyd1-t4F each carrying a *Bgl*II site (Table 1). All PCR products were digested with *Bgl*II/*Nco*I and introduced into *Bgl*II/*Nco*I linearized pET29b–eGFP (Ying and Feng, 2006), forming pET29b–Phyd1*x*–eGFP, in which *x* was the amplified fragment of −1798, −1290, −1179, −991 or −791 bp to drive the *eGFP* expression. The Phyd1*x*–eGFP cassette digested with *Xba*I was inserted into pAN52–bar (Ying and Feng, 2006) digested with *Xba*I and dephosphorylated in advance, generating binary plasmids pAN52–Phyd1*x*–eGFP–bar linking *eGFP* and *bar* [phosphinothricin (PPT) resistance gene] in the same transcriptional direction. For comparison, four more binary plasmids were constructed by replacing Phyd1*x* in the cassette respectively with the common promoter P*gpdA* and the 1290 bp fragment, in which the binding domain of one of three TFs (StuA, Mat-Mc and NIT_2_) was replaced with the *Hin*dIII restriction site using paired primers (Table 1). All DNA samples were cloned into pGEM-T-Easy (Promega, Madison, WI, USA) for sequencing at Invitrogen (Shanghai, China).

Each of the nine binary plasmids was inserted into the Bb2860 genome via blastospore transformation (Ying and Feng, 2006). Transgenic colonies grown on Czapek's plates containing 200 μg PPT per millilitre were examined via PCR with eGFP-F/R primers (Table 1) or under fluorescence microscope to confirm the presence of integrated *eGFP*. Three positive transformants arbitrarily taken from each of the *eGFP* constructs were grown on cellophane membranes overlaid on SDAY plates for 4 days at 25°C and 12:12 h (light : dark cycle) and then measured for their fluorescence intensities in a spectrophotometer FP-6500 (JASCO) at the excitation/emission wavelengths of 484/512 nm. Each measurement was expressed as RFI per milligram protein by deducting the background effect of wild-type strain (control) grown under the same conditions. Protein concentration in each of the extracts from the 4 day colonies was assessed using BCA (bicinchoninic acid) Protein Assay Kit (KeyGen, Nanjing, China) and bovine serum albumin (BSA) as standard. To monitor temporal pattern of *eGFP* expression, three transformants under a P*hyd1* fragment that maximized the *eGFP* expression were grown for 7 days on SDAY at the same regime and their RFI values were measured at 24 h interval. Colony samples taken during the growth period were visualized for fluorescence and bright images at the same wavelengths of excitation and emission under laser-scanning confocal microscope (Carl Zeiss, Germany).

### Expression of vip3Aa1 gene in *B. bassiana*

The eGFP element in pAN52–Phyd1-t1–eGFP–bar, in which Phyd1-t1 drove maximal eGFP expression, was replaced with the insecticidal protein gene *vip3Aa1* (GenBank ID: DQ539887.1). The resultant plasmid pAN52–P*hyd1-t1*–vip3Aa1–bar was then transformed into competent Bb2860 blastospores prepared with a previous method (Ying and Feng, 2006). The blastospores were spread on PPT-inclusive Czapek's plates for colony formation. Positive transformants were identified from the colonies by PCR with Vip-F/R and Bar-F/R (Table 1). Their *vip3Aa1* transcript levels in 4 day SDAY colonies grown at 25°C were compared with the transcript of an engineered strain (BbV28) expressing the same gene under P*gpdA* control (Qin *et al*., 2010). Reverse transcription of 5 μg of total RNA extracted from the colonies of each transformant was achieved using PrimeScript™ RT reagent kit (Takara, Japan). Synthesized cDNA (10 ng μl^−1^) was used as template for qRT-PCR with qVip-F/R and q18S-F/R (Table 1). The expression of the fungal 18S rRNA was used as internal standard. The level of *vip3Aa1* transcript in each cDNA sample was assessed using the 2^-ΔΔCt^ method (Livak and Schmittgen, 2001). The ratio of an estimate from each transformant over that from BbV28 was defined as the relative expression level of *vip3Aa1*.

Five transformants selected by qRT-PCR were further compared with BbV28 through elisa to assess the levels of the target protein (vip3Aa1) expressed in mycelia and conidia from SDAY colonies grown for 4 and 7 days respectively. Protein extracts from the mycelia and conidia were prepared as described previously (Qin *et al*., 2010). Three 100 μl aliquots (replicates) of each extract diluted with coating buffer (0.05 M carbonate, pH 9.6) were pipetted onto 96-well plate for overnight incubation at 4°C, followed by washing with 0.15 M phosphate-buffered saline (PBS, pH 7.4). Subsequently, each well was added with 200 μl of blocking buffer (0.1% BSA in coating buffer) for 1 h blocking at 37°C and then with 100 μl of diluted vip3Aa1 polyclonal antibody for 1 h reaction at 37°C. After PBS washing, each well was loaded with pre-diluted horseradish peroxidase-conjugated goat anti-rabbit IgG (Sigma) for 1 h incubation at 37°C. After final PBS washing, each well was loaded with 100 μl of TMB (3,3′,5,5′-tetramethylbenzidine) Liquid-1 Component (Amresco, Solon, OH, USA) for 20 min reaction at 37°C in dark and then with 100 μl of 2 M H_2_SO_4_ to terminate the reaction. Finally, OD_450_ value was read from each well using Model 680 Microplate Reader (Bio-Rad, Hercules, CA, USA). For each transformant, the relative expression level of vip3Aa1 was estimated as the ratio of its reading over that from BbV28. A transformant (BbHV8) expressing the highest level of the target protein was selected for assays below.

The presence or absence of vip3Aa1 in the mycelia and conidia of BbHV8, BbV28 and Bb2860 (negative control) was detected by Western blotting with the polyclonal antibody of vip3Aa1 (Qin *et al*., 2010). The density of vip3Aa1 molecules expressed in aerial conidia of each strain was estimated by immunogold localization. Briefly, conidia were fixed, dehydrated and embedded in pre-cooled resin of Lowicryl K4M (Plano, Wetzlar, Germany) under UV light of 360 nm wavelength. The final resin pyramids were cut into sections of 50−70 nm and mounted on 200-mesh Bioden Meshcement (Oken Shoji, Tokyo, Japan) coated with nickel grids (TAAB Laboratories, Berkshire, UK). The obverse side of the grids was treated for 30 min with the solution of 50 mM PBS (pH 7.2), 1% BSA, 0.02% PEG-20000, 100 mM NaCl and 0.1% NaNO_3_ and then incubated with 150 × dilution of the rabbit antibody for 1 h and then with 100 × dilution of 10 nm colloidal gold goat anti-rabbit IgG (Sigma) for 1 h. The ultrathin sections were finally contrasted with 1% uranyl acetate for 12 min and visualized under transmission electron microscope. For each strain, the 10 nm colloidal gold particles (targeted molecules) were counted from each of five sections labelled by the two antibodies and the mean density of labelled particles was estimated as the number of particles per unit area (μm^2^) based on the count in the total area of each ellipsoidal section.

### Bioassays for insecticidal activities

Bb2860, BbV28 and BbHV8 were incubated on steamed rice in Petri dishes (15 cm diameter) at 25°C for 7 days, followed by drying under ventilation at 33°C for 24 h. Aerial conidia were harvested from the rice cultures through a vibrating sieve, vacuum-dried to ∼ 5% water content at ambient temperature and then stored in glass tubes at −20°C for use within 3 months.

Aerial conidia of each strain was suspended in 0.02% Tween 80 and standardized to the concentration of 2 × 10^7^ conidia per millilitre. Three aliquots (replicates) of 1 ml each conidial suspension were separately sprayed onto lotus leaf discs (∼ 13 cm diameter) from the nozzle of Automatic Potter Spray Tower (Burkhard Scientific, Uxbridge, Middx, UK) at the working pressure of 0.7 kg cm^−2^. Each leaf disc under the spray harboured 30−40 *S. litura* larvae (instar II, III or IV). This experimental design was chosen to mimic field spray conditions, allowing the sprayed conidia to attach to cuticle for normal infection and to be ingested by the larvae for *per os* infection. Under each spray, the concentration of the conidia deposited onto the larvae and/or leaf disc was determined using five microscopic counts from a glass coverslip placed on the leaf disc. After spray, all larvae on the leaf discs were reared in Petri dishes (15 cm diameter) at 25°C and 12:12 h for up to 8 days. The leaf residues were replaced every 1 or 2 days with fresh leaf discs sprayed as above in advance. Three sprays of 1 ml 0.02% Tween 80 were used as blank controls in each bioassay. Larval mortality in each dish was examined daily during the period. The larvae killed by each strain were incubated at saturated humidity for 3−5 days. Cadavers with typical fungal outgrowths were considered to have died of normal fungal infection. Otherwise, their deaths were attributed to the action of vip3Aa1 released from ingested conidia (Qin *et al*., 2010).

To confirm further the *per os* insecticidal activity of the expressed vip3Aa1 to *S. litura* larvae, glass tubes filled with the concentrated conidial suspension (10^8^ conidia per millilitre) of each fungal strain were exposed to water bath at 42°C for 3 h to inactivate the conidia in the suspension. After the exposure, conidial viability in each suspension was evaluated using three 100 μl samples, which were spread onto SDAY plates for 24 h germination at 25°C. Each heat-treated suspension was sprayed onto lotus leaf discs for ingestion by the second-instar larvae of *S. litura* using the same method as above.

All time-mortality observations from the bioassays were analysed using the Probit Procedure in DPS software (Tang and Feng, 2007), generating LT_50_ estimates and associated 95% CIs as virulence indices of the tested fungal strains against *S. litura* larvae.
